# 1805. Tennessee’s Drug Diversion Investigation Team: A Collaborative Approach to a Growing Public Health Problem

**DOI:** 10.1093/ofid/ofad500.1634

**Published:** 2023-11-27

**Authors:** Callyn Wren, Christopher D Evans, Simone Godwin, Ashley Gambrell, Dipen Patel, Michelle Nation, Pamela Talley, Christopher Wilson

**Affiliations:** Tennessee Department of Health, Nashville, Tennessee; Tennessee Department of Health, Nashville, Tennessee; Tennessee Department of Health, Nashville, Tennessee; Tennessee Department of Health, Nashville, Tennessee; HAI/AR, Tennessee Department of Health, Nashville, Tennessee; TN Department of Health, Nashville, Tennessee; Tennessee Department of Health, Nashville, Tennessee; Tennessee Department of Health, Nashville, Tennessee

## Abstract

**Background:**

Injection drug use using nonsterile equipment can lead to transmission of viral, bacterial, and fungal infections. Frontline healthcare workers (HCW) are at high risk for substance use disorder due to unprecedented job stress and access to injectable controlled substances. The Tennessee Department of Health (TDH) developed a collaborative investigative process to determine the risk of bloodborne pathogen (BBP) transmission from licensed HCWs engaging in drug diversion. This program recommends public health action and provides consultation to improve drug diversion programs.

**Methods:**

In 2019 TDH formed a drug diversion investigation team (DDIT) consisting of pharmacists, epidemiologists and medical directors from the HAI and HIV/STI/Viral Hepatitis programs. The DDIT responds to notification by the Health-Related Boards (HRB) of a licensed HCW under investigation for diversion of injectable products. The DDIT interviews the investigator and meets the facility drug diversion program to review drug diversion policies and processes. Based on the suspected method(s) and, if known, the individual’s Hepatitis B/C and HIV status, recommendations are made regarding the need for patient notification and testing.

**Results:**

From 2020–2022 the DDIT received notification of 49 licensed HCWs under investigation for diversion of injectable products. Patient notification and testing was recommended in seven facilities for CDC Category A infection control breaches; in two cases, later HCW testing negated the need for further action. Among the 34 facilities queried, only five (14.7%) had existing policies for for-cause BBP testing. Other recommendations to improve diversion programs include infection prevention participation and releasing “not eligible for rehire” status to other facilities.

**Conclusion:**

The TDH DDIT facilitates communication with HRB on reported cases of injectable drug diversion. Joint investigations with facilities raise awareness of the risk of BBP transmission and improve facility diversion programs. Tennessee facilities are adding for-cause BBP testing to their investigation procedures. The TDH DDIT model receives mostly positive responses from facility and health system drug diversion teams and may be considered by other public health jurisdictions.

**Disclosures:**

**All Authors**: No reported disclosures

